# Anaplastic Lymphoma Kinase-Positive Primary Lung Adenocarcinoma Presenting With Pericardial Effusion and Tamponade in a COVID-19 Patient: A Case Report

**DOI:** 10.7759/cureus.19127

**Published:** 2021-10-29

**Authors:** Ateeb Ur Rahman, Olufunmilayo Folaranmi, Vernon Chan, Amna Chaudary

**Affiliations:** 1 Internal Medicine, WellSpan York Hospital, York, USA

**Keywords:** alk-positive lung adenocarcinoma, covid-19, pulmonary embolus, deep venous thrombosis, pericardial effusion

## Abstract

Primary lung cancer usually presents in older adults with a smoking history. However, there is an estimated incidence of 15-20% among men who have never smoked. The diagnosis of lung malignancy can often be incidental. Moreover, cardiac tamponade can be an initial presentation of malignancy, especially lung cancer, as these are the most common tumors that involve the pericardium.

Here, we present a 54-year-old male with no known medical history presented with dyspnea on exertion. He was found to have a large pericardial effusion with tamponade physiology on a bedside echocardiogram. He was also found to have bilateral deep vein thrombosis and pulmonary embolism on admission. The patient underwent an emergent pericardiocentesis due to hemodynamic compromise, and pericardial fluid cytology suggested adenocarcinoma with lung primary. Subsequently, gene testing revealed anaplastic lymphoma kinase-positive adenocarcinoma of the lung. The patient was discharged home with close oncology follow-up. It is imperative to recognize malignant pericardial effusion as one of the causes of dyspnea. Emergent pericardiocentesis may be needed in case of hemodynamic compromise and tenuous clinical status.

## Introduction

Lung cancer is the most common cancer worldwide and is responsible for most cancer deaths [1] according to the World Health Organization. Adenocarcinoma is the most common lung cancer among nonsmokers [2,3]. However, young people and light smokers have also been reported to have adenocarcinoma, especially in association with anaplastic lymphoma kinase (ALK) [4]. While it is not uncommon for lung cancer, breast cancer, lymphomas, leukemia, and melanomas to involve the pericardium [5], it is rare for nonsmall cell lung cancer (NSCLC) to present with pericardial effusion and cardiac tamponade, which are generally associated with poor prognosis [6-8].

In this case report, we present a case of lung adenocarcinoma with an initial diagnosis of pericardial effusion with tamponade in a coronavirus disease 2019 (COVID-19)-positive patient.

## Case presentation

A 54-year-old Caucasian male with no significant medical history presented to the emergency room (ER) with progressively worsening shortness of breath over two weeks. A review of systems was unremarkable except for bilateral leg swelling that was worse on the left side, which he attributed to a sprained left ankle a few weeks prior. Social history revealed that the patient was a nonsmoker. He was diagnosed with COVID-19 a few weeks prior to his ER visit.

On presentation, the patient’s initial vital signs were blood pressure of 99/73 mmHg, heart rate of 129 beats per minute, respiratory rate of 28 breaths per minute, temperature of 36.8°C (98.3°F), and SpO_2_ of 91% on room air. He had mild respiratory distress on physical examination. Lung auscultation revealed few scattered crackles in the lung bases. Additionally, he was tachycardic, with an irregular rhythm. There was bilateral lower extremity swelling with the left greater than the right along with associated mild left calf tenderness.

**Figure 1:**
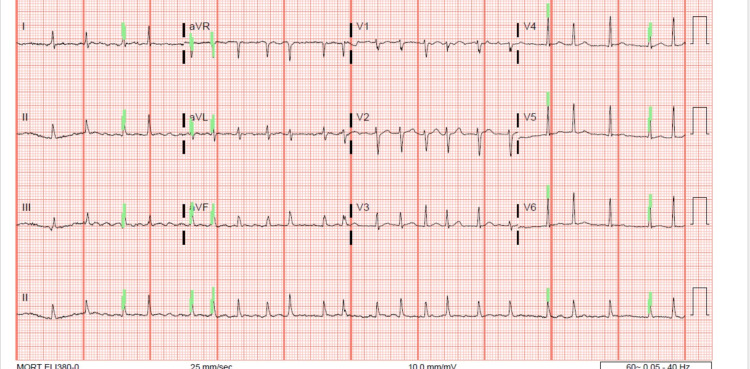
EKG exhibiting atrial fibrillation with electrical alternans. EKG: electrocardiogram

An initial electrocardiogram (EKG) showed new-onset atrial fibrillation (Figure 1) with a rapid ventricular response and electrical alternans.

**Figure 2:**
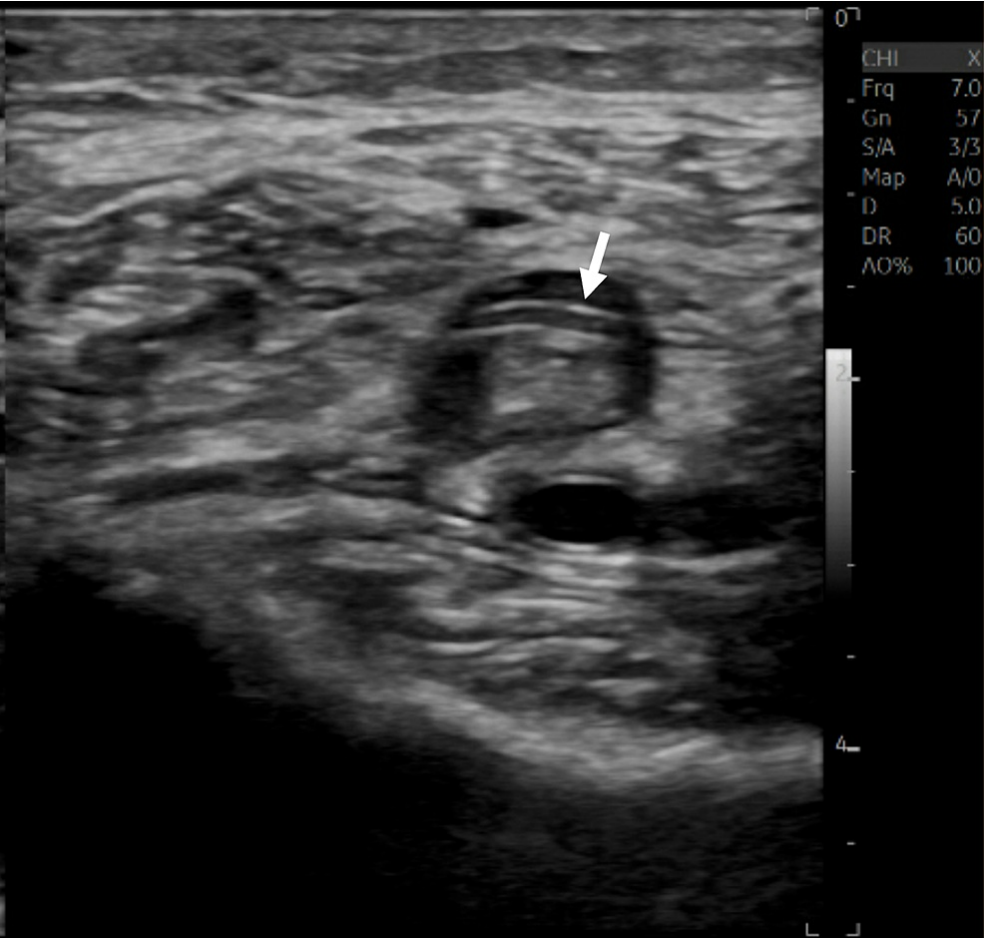
DVT in the left popliteal vein. DVT: deep vein thrombus

Significant abnormal laboratory findings were as follows: troponin-I of 0.04 ng/mL, B-type natriuretic peptide of 378 pg/mL, D-dimer of 19 mg/L, the international normalized ratio of 1.3, leukocytosis of 21,000/µL, and lactate of 6.0 mmol/L. A repeat COVID-19 test was also positive. Furthermore, lower extremity ultrasound showed right lower extremity deep venous thrombosis (DVT) involving the right posterior tibial vein and peroneal vein, as well as left lower extremity DVT (Figure 2) involving the left posterior tibial vein, peroneal vein, and popliteal vein.

**Figure 3:**
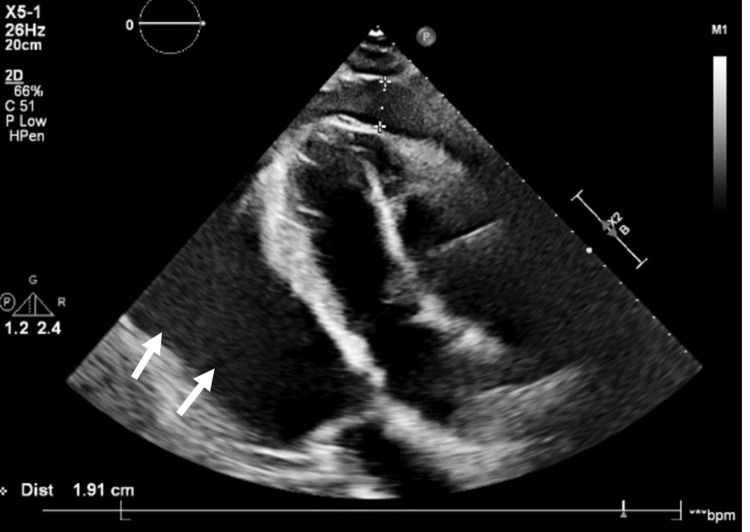
Large circumferential pericardial effusion surrounding the heart, as seen in the parasternal long axis.

A bedside echocardiogram revealed a large pericardial effusion (Figure 3). Although there was right ventricular filling, on cardiac monitoring, electrical alternans was noted. Due to concern for cardiac tamponade, cardiology was consulted, and the patient underwent emergent pericardiocentesis with a possible diagnosis of post-COVID myocarditis/pericarditis. Hemorrhagic pericardial fluid (2 L) was removed.

**Figure 4:**
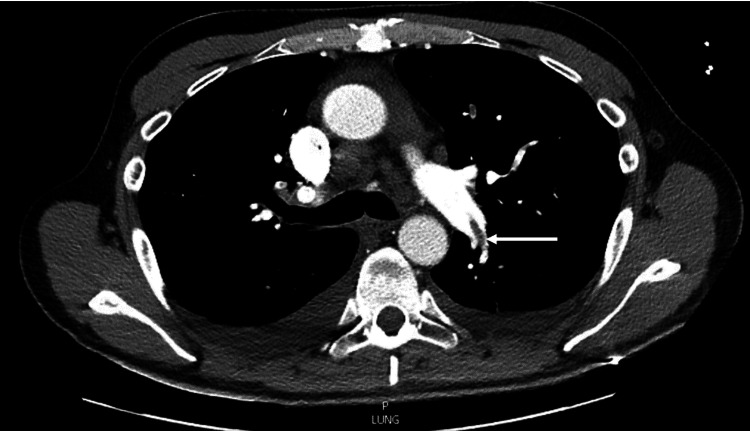
Filling defect in the left central pulmonary artery.

The patient was admitted to the COVID-19 critical care unit directly from the catheterization lab for closer hemodynamic monitoring. Due to his persistent hypoxia, a CT angiogram of the chest was done which revealed bilateral acute pulmonary emboli (Figure 4) with right ventricular strain.

**Figure 5:**
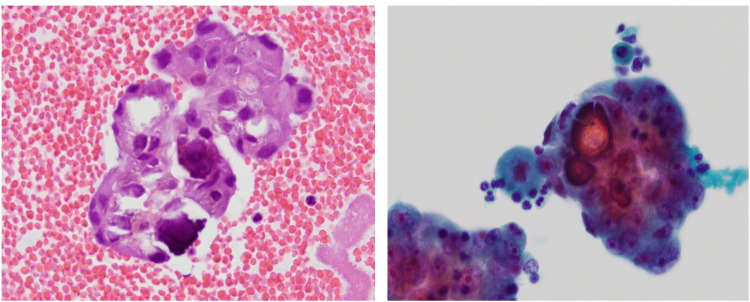
Pericardial fluid tumor fragment.

**Figure 6:**
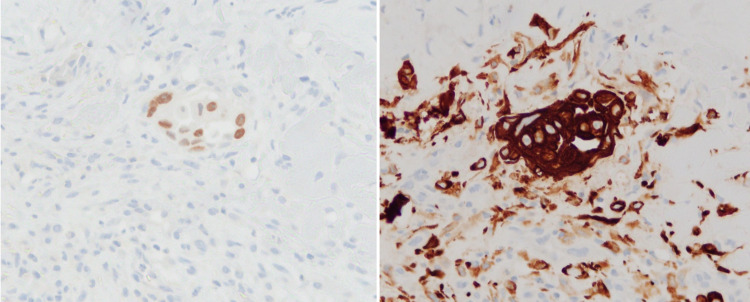
Pericardial tissue carcinoma with TTF-1 stain (left) and EPCAM stain (right). TTF: thyroid transcription factor 1; EPCAM: epithelial cell adhesion molecule

The patient underwent a pericardial drain placement with subsequent placement of a pericardial window. Cytology of the pericardial biopsy was consistent with adenocarcinoma of lung origin. Cytology also showed high expression of programmed death-ligand 1. Immunostains demonstrated the cells to be positive for BER-EP4 and thyroid transcription factor 1 (patchy). These findings indicated a metastatic carcinoma likely compatible with adenocarcinoma of lung origin (Figures 5, 6). The pathology report from his pericardial biopsy showed metastatic adenocarcinoma likely from the lung. Subsequently, next-generation sequencing was done, which was positive for ALK mutation. Staging scans were done in which CT of the abdomen and pelvis showed a 2.2 × 2.8 × 2.2 cm lytic lesion in the S1 vertebra and a 2.8 × 3.6 × 4.1 cm lesion in the posterior trochanteric region of the left femur. Moreover, retroperitoneal and mediastinal lymphadenopathy was also noted. However, an MRI of the brain was normal for his age. The patient was discharged home after improvement in clinical status with oncology follow-up as an outpatient. He is currently on active treatment with alectinib due to positive ALK mutation and is receiving external beam radiation therapy for the left femur metastases.

## Discussion

NSCLCs account for approximately 75% of all types of lung cancers comprising three major histologic types, namely, squamous cell carcinoma, adenocarcinoma, and large cell carcinoma. Adenocarcinoma constitutes 40% of all lung cancers worldwide [9]. More than one-third of the patients who have lung adenocarcinomas are diagnosed at stage 4 [10]. Oncogene ALK (EML4-ALK) fusion genes were discovered in 2007. Unlike other genotypes, the ALK-positive type can manifest with different histological subtypes on presentation [4]. ALK gene reassortments affect only approximately 4% of all lung cancers. The frequency of these mutations is more common in adenocarcinoma, nonsmokers, and is concomitant with EGFR/KRAS mutations [11].

The most frequent causes of pericardial effusion are idiopathic (26%), malignancy (25%), and iatrogenic (20%) [12]. In malignancy, lung cancer tops the list. Breast cancer is second on the list with 18% metastatic spread to the pericardium [12]. Although uncommon, cardiac involvement maybe the first presenting sign of a malignancy in the form of overt or insidious pericardial tamponade [13]. Cardiac involvement is seen in almost one-tenth of all cancer patients, and one-third of the patients die as a consequence [14]. Doebele et al. studied mutational analysis and patterns of metastatic spread in NSCLC and reported that ALK gene rearrangements had more metastatic pericardial and pleural spread compared to other mutations [15].

Studies have suggested a median survival of 12-15 months after a malignant pericardial effusion is diagnosed, especially in lung cancer compared to other solid tumors [16]. Moreover, cancer-related pericardial effusion is associated with worse outcomes compared to noncancerous pericardial effusion. Increased rates of recurrent effusions, pericardiocentesis, and pericardial surgery have also been reported [17].

Our patient was ALK-positive which suggests a good response to ALK inhibitors. Identifying driver genes is essential for the targeted treatment of lung adenocarcinomas. Tyrosine kinase inhibitors such as crizotinib have been shown to be effective as a treatment option in ALK-positive adenocarcinoma [4]. Despite treatment, overall five-year survival for all stages of adenocarcinoma is 15% [10]. The overall survival in metastatic ALK-positive adenocarcinoma is also very poor [18].

## Conclusions

Presumably rare, we reported a case of a primary lung adenocarcinoma that presented with pericardial effusion in a COVID-19-positive patient. While a patient’s presentation might easily check the box for an obvious diagnosis, it might be a camouflage for something perhaps more sinister which changes the trajectory of patient management when unveiled. In this case, COVID-19 was a very close masquerading differential. Having a very broad list of potential differential diagnoses is crucial.

A thorough physical examination, important and timely imaging, and prompt surgical intervention with appropriate escalation of care will improve the overall outcomes and quality of life in these patients. Early detection of lung adenocarcinoma associated with ALK is very important for survival as most of these cases present at an advanced stage. More studies are needed for a better understanding of the pathophysiology of adenocarcinoma. This improved knowledge would also lead to updated guidelines and recommendations for prompt treatment to improve the overall prognosis of these patients.

## References

[1] Wakelee HA, Chang ET, Gomez SL (2007). Lung cancer incidence in never smokers. J Clin Oncol.

[2] Rivera GA, Wakelee H (2016). Lung cancer in never smokers. Adv Exp Med Biol.

[3] Samet JM, Avila-Tang E, Boffetta P, Hannan LM, Olivo-Marston S, Thun MJ, Rudin CM (2009). Lung cancer in never smokers: clinical epidemiology and environmental risk factors. Clin Cancer Res.

[4] Kim H, Chung JH (2015). Overview of clinicopathologic features of ALK-rearranged lung adenocarcinoma and current diagnostic testing for ALK rearrangement. Transl Lung Cancer Res.

[5] Buzaid AC, Garewal HS, Greenberg BR (1989). Managing malignant pericardial effusion. West J Med.

[6] Li BT, Pearson A, Pavlakis N (2014). Malignant cardiac tamponade from non-small cell lung cancer: case series from the era of molecular targeted therapy. J Clin Med.

[7] Wang PC, Yang KY, Chao JY, Liu JM, Perng RP, Yen SH (2000). Prognostic role of pericardial fluid cytology in cardiac tamponade associated with non-small cell lung cancer. Chest.

[8] Cullinane CA, Paz IB, Smith D, Carter N, Grannis FW Jr (2004). Prognostic factors in the surgical management of pericardial effusion in the patient with concurrent malignancy. Chest.

[9] Myers DJ, Wallen JM (2021). Lung adenocarcinoma. https://pubmed.ncbi.nlm.nih.gov/30137862/.

[10] Zhu QG, Zhang SM, Ding XX, He B, Zhang HQ (2017). Driver genes in non-small cell lung cancer: characteristics, detection methods, and targeted therapies. Oncotarget.

[11] Solomon B, Varella-Garcia M, Camidge DR (2009). ALK gene rearrangements: a new therapeutic target in a molecularly defined subset of non-small cell lung cancer. J Thorac Oncol.

[12] Strobbe A, Adriaenssens T, Bennett J (2017). Etiology and long-term outcome of patients undergoing pericardiocentesis. J Am Heart Assoc.

[13] Shapiro LM (2001). Cardiac tumours: diagnosis and management. Heart.

[14] Maisch B, Ristic A, Pankuweit S (2010). Evaluation and management of pericardial effusion in patients with neoplastic disease. Prog Cardiovasc Dis.

[15] Doebele RC, Lu X, Sumey C (2012). Oncogene status predicts patterns of metastatic spread in treatment-naive nonsmall cell lung cancer. Cancer.

[16] El Haddad D, Iliescu C, William WN, Mouhayar E (2015). Survival outcomes of cancer patients with pericardial effusions. J Clin Oncol.

[17] Gornik HL, Gerhard-Herman M, Beckman JA (2005). Abnormal cytology predicts poor prognosis in cancer patients with pericardial effusion. J Clin Oncol.

[18] Tsimafeyeu I, Moiseenko F, Orlov S (2019). Overall survival of patients with ALK-positive metastatic non-small-cell lung cancer in the Russian Federation: nationwide cohort study. J Glob Oncol.

